# MicroRNA-133b Dysregulation in a Mouse Model of Cervical Contusion Injury

**DOI:** 10.3390/ijms25053058

**Published:** 2024-03-06

**Authors:** James Young Ho Yu, Thomas C. Chen, Camelia A. Danilov

**Affiliations:** 1 Department of Neurological Surgery, University of Southern California, 1200 N State St., Suite 3300, Los Angeles, CA 90033, USA; james.yu@med.usc.edu (J.Y.H.Y.); thomas.chen@med.usc.edu (T.C.C.); 2 Department of Neurological Surgery, University of Southern California, 2011 Zonal Ave., Los Angeles, CA 90089, USA

**Keywords:** microRNA133b, spinal cord injury, spine trauma, scar tissue, cervical spine, motor cortex

## Abstract

Our previous research studies have demonstrated the role of microRNA133b (miR133b) in healing the contused spinal cord when administered either intranasally or intravenously 24 h following an injury. While our data showed beneficial effects of exogenous miR133b delivered within hours of a spinal cord injury (SCI), the kinetics of endogenous miR133b levels in the contused spinal cord and rostral/caudal segments of the injury were not fully investigated. In this study, we examined the miR133b dysregulation in a mouse model of moderate unilateral contusion injury at the fifth cervical (C5) level. Between 30 min and 7 days post-injury, mice were euthanized and tissues were collected from different areas of the spinal cord, ipsilateral and contralateral prefrontal motor cortices, and off-targets such as lung and spleen. The endogenous level of miR133b was determined by RT-qPCR. We found that after SCI, (a) most changes in miR133b level were restricted to the injured area with very limited alterations in the rostral and caudal parts relative to the injury site, (b) acute changes in the endogenous levels were predominantly specific to the lesion site with delayed miR133b changes in the motor cortex, and (c) ipsilateral and contralateral hemispheres responded differently to unilateral SCI. Our results suggest that the therapeutic window for exogenous miR133b therapy begins earlier than 24 h post-injury and potentially lasts longer than 7 days.

## 1. Introduction

Acute traumatic spinal cord injury (SCI) in the cervical spine is a condition commonly associated with falls and high-impact traumas, often leading to severe and lasting neurologic disabilities despite medical and surgical interventions [1]. These neurologic disabilities range from motor and sensory deficits to bowel, bladder, and sexual dysfunction, tremendously impairing quality of life and productivity [2,3]. Despite the rising global incidence of SCI, the management of acute SCI remains limited to surgical decompression and stabilization of the spine to prevent secondary injuries, as well as increasing the blood pressure to augment perfusion of the spinal cord. The current standard of care does not involve directed therapies to address the initial neural injury to the spinal cord. The challenges of achieving primary neuro-restoration in SCIs include poor regenerative properties of the spinal cord itself and time sensitivity for administering therapeutic interventions [4].

In order to advance targeted therapies for SCIs, recent works in the literature have examined the neuroprotective mechanism of microRNAs (miRs). MicroRNAs are a class of short (~22 nucleotides) endogenous non-coding regulatory RNA molecules, which play important roles in post-transcriptional gene regulation [5,6,7]. The major mechanism of gene regulation is to bind target mRNA and initiate either translational repression or mRNA degradation [8,9].

The expression of neuroprotective miRs in animal models of SCIs promotes resistance to apoptotic cell death, demyelination, and scar formation by altering the perilesional microenvironment and composition of the extracellular matrix [10]. Moreover, existing evidence indicate that numerous miRs have been involved in neurogenesis, axon growth and neuronal maturation in the central nervous system (CNS) [6,11].

miR133b is among the miRs that have relevance for spinal cord regeneration. A link between elevated miR133b expression level, axonal regeneration, and recovery of motor function was first demonstrated in a spinal cord injury model in zebrafish, an excellent model of spontaneous regeneration [12]. Our published data have established that once-a-day intravenous delivery of miR133b with Argonaute 2 (Ago2, an miR stabilizer) for 3 consecutive days starting 24 h after a spinal cord contusion targeted the microenvironment at the injury site via (i) downregulating gene expression for extracellular matrix molecules, such as collagen type 1 alpha 1 (*Col1a1*) and tenascin N (*Tnn*), at the lesion scar, (ii) decreasing inflammation and lesion scar volume, leading to enhanced forelimb grip function in injured mice [13]. Recently, we demonstrated that intranasal co-administration of miR133b with NEO100, a highly purified GMP-manufactured perillyl alcohol, resulted in better cellular uptake of miR133b at the lesion and improved healing of the injured spinal cord [14].

The role of miR133b in functional recovery after SCI was further supported by another group who reported that lentiviral delivery of miR133b to the injury site immediately after thoracic compression injury led to restoration of locomotor function through a mechanism that involved RhoA, xylosyltransferase-1, ephrin receptor A7, and purinergic receptor P2X ligand-gated ion channel 4 downregulation [15].

The involvement of miR133b in promoting neuroplasticity and functional recovery has also been illustrated in a stroke model in rats, where miR133b was delivered via exosome-enriched extracellular particles [16]. Additionally, it has been reported that stereotaxic injection of miR133b into the hippocampus protects neurons against inflammation and apoptotic cell death in a model of depression in rats [17], and lateral cerebroventricular injection of miR133b attenuates isoflurane-induced learning and memory impairments in rats [18], highlighting the neuroprotective role of miR133b against cell death.

While our findings are consistent with other works that have demonstrated the beneficial effect of miR133b in healing injured neural tissue, there are limited data on the kinetics of endogenous miR133b levels in response to SCI.

The overall goals of the present study were: (a) to examine the effect of contusive-type injury on the endogenous level of miR133b in the spinal cord, including the lesion site and rostral/caudal areas in close proximity to the injury; (b) to evaluate the degree to which a contusive injury at the spinal cord affects the endogenous miR133b level in the prefrontal ipsilateral and contralateral cortices; and (c) to determine the effect of SCI on miR133b level in off-targets, such as the lung and spleen. In this study, we used a mouse model of a moderate unilateral contusive injury at the fifth cervical (C5) level. This injury model was suitable for our investigations for several reasons: (a) high clinical relevance given that most spinal cord injuries in humans occur at the cervical level (60–75%) as a contusive type injury, where the dura remains intact and incomplete bruising partially spares neural tissue of the spinal cord; (b) a unilateral injury allowed us to determine the effect of SCI on the sensorimotor cortex, as well as the response between ipsilateral and contralateral hemispheres [19].

Here, we first determined the dynamics of the endogenous level of miR133b in the lesion area between 30 min and 7 days after an injury. Then, we investigated to what extent miR levels are affected in proximal and distal parts of the injury. Next, we determined the correlation between changes in miR133b levels in the contused spinal cord and brain. Finally, we assessed the level of miR133b in off-targets with the purpose of understanding whether a local injury at the spinal cord level has a systemic effect on other organs, such as lung and spleen. Understanding the behavior of endogenous miR133b in lung and spleen in response to SCI was of particular interest because our previous work highlighted intranasal and intravenous routes of delivery as potential strategies for clinical administration of exogenous miR133b.

The study presented here opens a new door for understanding the dynamics of endogenous miR133b at the site of the lesion and in neighboring segments of the spinal cord proximal and distal to the lesion, with the hope of optimizing the time of intervention, route of administration, and duration of miR133b therapy.

## 2. Results

We previously reported the efficacy of miR133b in healing the injured spinal cord when administered either intravenously [13] or intranasally [14] 24 h after injury. To better understand the dynamics of miR133b in a contusive type of injury, in this study we aimed to determine the changes in the endogenous level of miR133b at (a) the lesion site, (b) proximal and distal sites of the lesion, (c) pre-frontal cortex, and (d) off-targets, such as spleen and lungs, at different time points after SCI.

### 2.1. Changes in the Endogenous Level of miR133b in the Contused Spinal Cord

**Figure 1:**
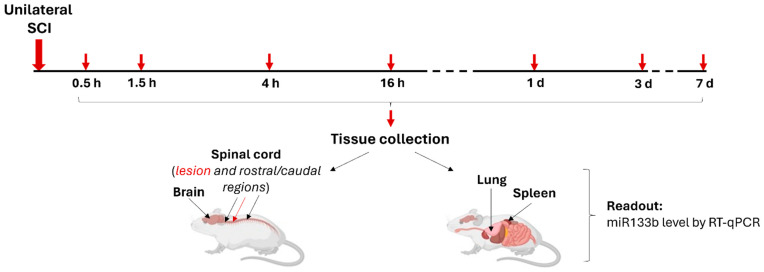
Schematic diagram of the research study design. Mice received unilateral spinal cord injury at the cervical 5th level. At different time points between 0.5 h and 7 days post-injury, mice were euthanized followed by the tissue harvesting as follows: spinal cord (lesion area, medulla and thoracic segments), brain (ipsilateral and contralateral hemispheres), lung and spleen. The level of miR133b in different tissue samples was determined by RT-qPCR. H = hours and d = days post-injury.

We first determined the dynamics of the endogenous level of miR133b in the lesion scar between 30 min and 7 days after injury. After receiving a moderate unilateral contusion injury, mice were euthanized and the contused spinal cord was collected as described in Methods. miR133b level was measured by RT-qPCR. Figure 1 represents the experimental design for this study with the indicated time points for tissue collection and miR133b quantification.

**Figure 2:**
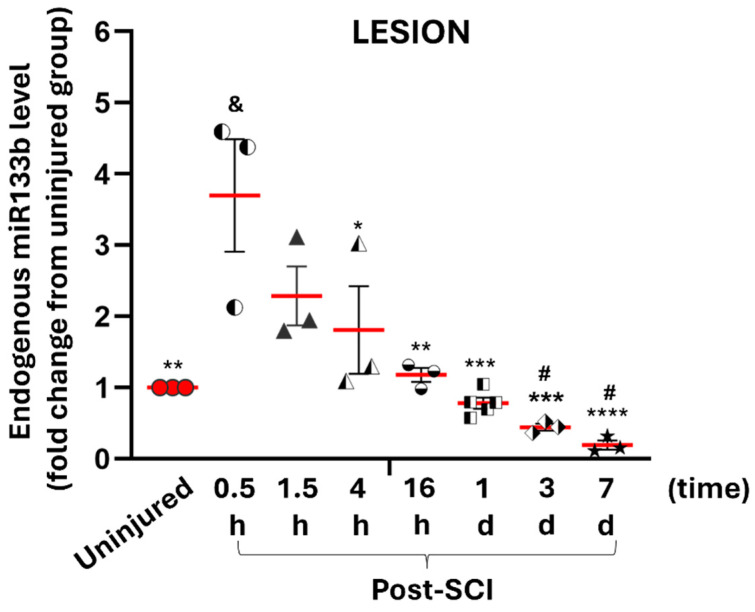
The effect of spinal cord injury on the endogenous level of miR133b at the site of injury. Between 0.5 h and 7 days after receiving a cervical contusion injury, mice were euthanized and spinal cords that included the lesion scar were collected and processed for microRNA isolation. The level of miR133b was assessed by RT-qPCR analysis. One-way ANOVA was used for analysis, with Tukey’s post hoc test for multiple comparisons test. * *p* < 0.05, ** *p* < 0.01, *** *p* < 0.001, **** *p* < 0.0001 when compared to 0.5 h injury group; ^#^ *p* < 0.05 when compared to 1.5 h injury group; ^&^ *p* < 0.0004 when compared to uninjured group. The circles, triangles, squares, and stars in the graph represent the individual mouse miR level per group. The red line shows the mean with SEM.

Figure 2 shows relative miR133b levels in the lesion scar expressed as fold change compared to levels measured in the cervical (C4–C6) segment isolated from uninjured mice. Acutely, 30 min after injury, there was a statistically significant 3.7-fold increase in the endogenous level of miR133b compared to the uninjured group (*p* < 0.0001). After the initial increase, the miR level started to decrease gradually and by 4 h there was a 50% decrease (1.8-fold decrease). At 1-day post-SCI, the decrease was more pronounced with a 78% decrease (0.8-fold) compared to the 30 min time point (*p* < 0.001). Interestingly, the endogenous level of miR was still affected at the lesion site 7 days later, reaching a 94.6% decrease (0.2-fold) (*p* < 0.0001). These data suggest that a contusive injury affects the endogenous level of miR133b at the site of impact and the effect is maintained up to seven days post-injury.

### 2.2. Endogenous Level of miR133b at Sites Proximal and Distal to the Injury

Having determined the changes in miR133b expression at the injury site, we next investigated whether the endogenous level is affected to the same extent in other segments of the spinal cord or whether the changes are exclusively related to the site of injury. For this purpose, we measured the miR level in two parts of the spinal cord: (a) medulla–1st cervical (C1) segment (rostral part) and (b) 5th thoracic (T5)–7th thoracic (T7) segments (caudal part).

**Figure 3:**
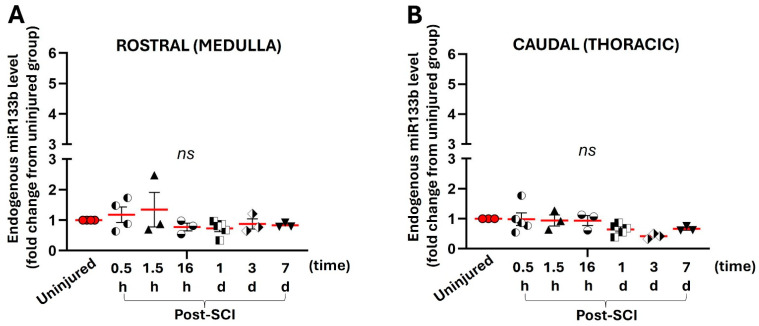
The effect of spinal cord injury on the endogenous level of miR133b in the rostral and caudal parts of the spinal cord. The rostral and caudal parts were collected from mice that received cervical contusion injury at the indicated time points. Panel (**A**) represents the level of miR measured in the medulla–cervical 1st (C1) segment (rostral part). Panel (**B**) shows the level of miR in the thoracic 5th (T5) –thoracic 7th (T7) segment of the spinal cord (caudal part). One-way ANOVA was used for analysis, with Dunnett’s multiple comparisons test. Ns = not significant; *p* = 0.45 and F = 1.00 (rostral part) and *p* = 0.093 and F = 2.18 (caudal part). The circles, triangles, squares, and stars in the graph represent the individual mouse miR level per group. The red line shows the mean with SEM.

Figure 3A,B shows relative levels of miR133b in the rostral and caudal parts expressed as fold change compared to the levels measured in the corresponding parts isolated from uninjured mice. Cervical contusion injury did not significantly affect miR133b level in the medulla compared to the uninjured group at any time point of the experiment (Figure 3A) (*p* = 0.45 and F = 1). There was a trend towards a slight increase in miR level at 30 min (1.18-fold increase) and 1.5 h (1.35-fold increase) compared to the uninjured group (1-fold), but it was not statistically significant. Starting at 16 h post-injury, we found a small downward trend (0.78-fold decrease) in miR levels with no notable changes until the end of the study at 7 days (0.83-fold decrease) post-injury.

As shown in Figure 3B, the relative miR133b levels in the thoracic part were similar to the levels measured in the rostral part. Our data showed that cervical injury did not alter the endogenous level of miR133b in the thoracic cord at earlier time points, such as 30 min (1-fold) and 1.5 h (0.94-fold), compared to the values in the uninjured thoracic part (1-fold). As found in the rostral part, there was a trend toward decreased miR level after 1 day (0.65-fold decrease) that remained unchanged up to 7 days post-SCI (0.67-fold decrease) (*p* = 0.09 and F = 2.18). Taken together, the data suggest that a contusive injury has minimal effect on the endogenous level of miR133b at proximal and distal sites to the injury.

### 2.3. The Effect of SCI on the Endogenous Level of miR133b in the Prefrontal Cortex

We next aimed to determine the extent to which an injury at the cervical cord level changes the endogenous miR133b level in the brain. To assess this aim, we collected the prefrontal cortex from both ipsilateral and contralateral hemispheres in mice receiving a unilateral cervical cord contusion injury. Changes in miR133b levels in the brain were measured by RT-qPCR.

**Figure 4:**
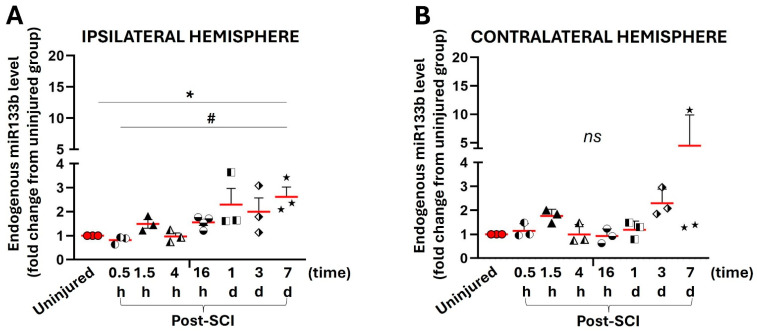
The effect of cervical contusion injury on the endogenous level of miR133b in the prefrontal cortex. Between 0.5 h and 7 days after receiving a unilateral contusion injury at the C5th level, mice were euthanized and pre-frontal cortices harvested and processed for miR isolation. The graphs represent the level of miR133b in the ipsilateral (panel (**A**)) and contralateral (panel (**B**)) hemispheres. One-way ANOVA was used for analysis, with Dunnett’s for multiple comparisons test. * *p* < 0.05 when compared to untreated group; ^#^ *p* < 0.05 when compared to 0.5 h group. The circles, triangles, squares, and stars in the graph represent the individual mouse miR level per group. The red line shows the mean with SEM.

Figure 4A,B shows relative levels of miR in the ipsilateral and contralateral hemispheres as fold change compared to the levels measured in the corresponding parts isolated from uninjured mice. At 30 min after the injury, there were no significant changes in miR133b level in either ipsilateral (0.8-fold change) (Figure 4A) or contralateral (1.15-fold change) (Figure 4B) hemispheres compared to the corresponding hemisphere isolated from the uninjured mice. There was a trend towards an increase in miR level at 1.5 h, such as a 1.5-fold increase ipsilaterally and 1.77-fold increase contralaterally, that remained unchanged until 16 h. In contrast to the miR levels detected in the lesion between 1 and 7 days post-injury, miR levels in the brain were rather high. At 7 days, the miR level measured in the ipsilateral hemisphere was significantly higher (2.62-fold increase) than the levels in both uninjured (*p* < 0.05) and 30 min (*p* < 0.05) groups. In the contralateral hemisphere, there was a trend towards an increase in miR level (4.47-fold increase) (*p* = 0.38 and F = 1.15). Altogether, the data indicate that contusive spinal cord injury affects the level of miR133b in the brain, but at later time points post-injury compared to the lesion site.

### 2.4. Correlation Studies between the Level of miR133b in the Contused Spinal Cord and Brain

To better understand the relationship between changes in the miR level (a) in the contused spinal cord and brain (pre-frontal cortex), and (b) in the ipsilateral and contralateral hemispheres, we ran a Pearson correlation coefficient analysis.

**Figure 5:**
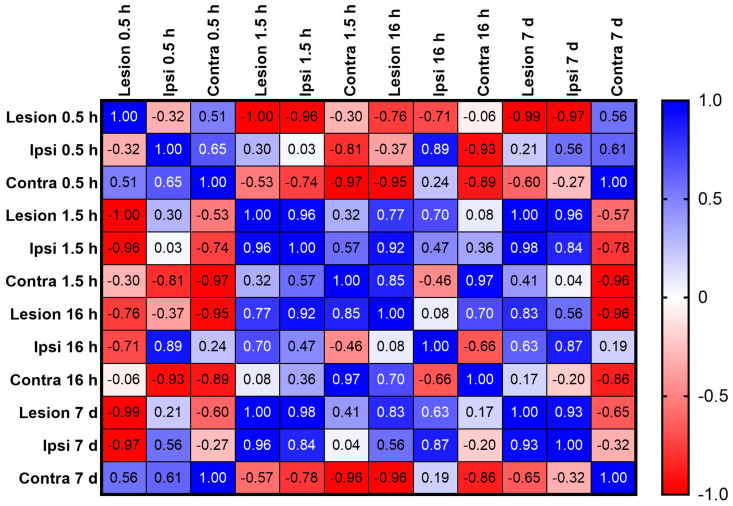
Heatmap of the Pearson correlation coefficient matrix between lesion and prefrontal cortex. The heatmap shows the correlation analysis (r values) between levels of miR133b measured in the lesion and ipsilateral and contralateral hemispheres. The r values were obtained by performing a Pearson correlation coefficient test.

Figure 5 shows the heatmap of the Pearson correlation coefficient matrix between the lesion and ipsi- and contralateral hemispheres at 0.5 1.5 h, 16 h, and 7 days post-injury. When we compared the levels of miR in the lesion with the levels in the brain, we found that shortly after SCI, there was a moderate to strong positive correlation with the miR levels in the contralateral side (r = 0.51) and a weak negative correlation with the levels in the ipsilateral part (r = −0.32). Ninety minutes later, the changes in the ipsilateral side became more pronounced and positively correlated in comparison to the ones in the lesion (r = 0.96), while the correlation in the contralateral hemisphere remained positive but slightly weaker (r = 0.32). Moreover, the heatmap of the Pearson correlation indicated that there was a moderate to strong correlation between the changes in miR level in both hemispheres (r = 0.57). At 16 h, there was a significant positive correlation between changes in miR level in the lesion and the ones in the contralateral side (r = 0.7) and a significant negative correlation between ipsilateral and contralateral parts (r = −0.66). At 7 days, the correlation analysis showed that there was a significant negative correlation between miR levels in the contralateral side and lesion (r = −0.65) and a moderate negative correlation between both hemispheres (r = −0.32). Based on these data, we can conclude that there is a correlation between changes in levels of miR133b in the contused spinal cord and motor cortex, as well as between ipsilateral and contralateral hemispheres with different strengths over a period of 7 days after the injury.

### 2.5. Endogenous Level of miR133b in Off-Targets after Cervical Contusion Injury

We further investigated the effect of cervical contusion in off-targets such as lungs and spleen. The levels of miR133b in these tissue samples were quantified as a fold change compared to the levels measured in the lungs and spleen isolated from mice without injury.

**Figure 6:**
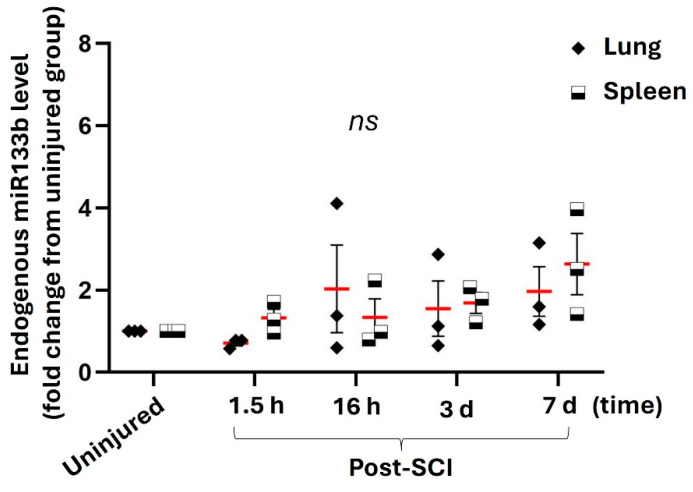
The endogenous levels of miR133b in the lung and spleen after cervical contusion injury. The graph represents the level of miR133b in off-targets at different time points following the injury. Two-way ANOVA was used for analysis, with Tukey’s for multiple comparison test. Ns = not statistically significant.

Figure 6 shows relative levels of miR133b in the lungs and spleen at different time points post-injury. Our data revealed that a cervical contusion injury did not contribute to significant changes in the level of miR in either lung or spleen compared to the endogenous levels of miR in normal off-target tissues (*p* = 0.58 and F = 0.6). Shortly after the injury, there was a slight increasing trend in the spleen (1.3-fold increase) when compared to the level in the uninjured tissue (1-fold change) and in the lung (0.7-fold change), respectively. Over the course of 3 days after injury, the levels of miR in both off-targets were similar, with a 1.6-fold change in lung vs. 1.7 in spleen. miR values remained quite similar between these tissues for up to 7 days, with a range of 2–2.5-fold change. Taken together, these data suggest that a contusive type of cervical cord injury has a minimal effect on the miR133b levels in lungs and spleen.

## 3. Discussion

### 3.1. Rationale for the Approach

We have previously reported that administration of miR133b, either intravenously [13] or intranasally [14], in combination with NEO100, a purified version of perillyl alcohol, targeted the microenvironment of the fibrotic scar, leading to improved healing and motor function in a mouse model of cervical contusion injury. While these studies highlighted the beneficial role of miR133b when administered 24 h after spinal cord injury, its local dynamics in the lesion, as well as in other areas of the spinal cord, are not well-known. Therefore, in this study, we developed a strategy to better understand (a) the effect of contusive injury on the endogenous level of miR133b in the spinal cord, (b) how miR changes at the injury site correlate with miR changes in the brain, and (c) the extent to which a spinal cord injury affects miR133b levels in off-targets, such as the lung and spleen.

Our methodology was tested in a mouse model of SCI, specifically a moderate unilateral cervical contusion, which is clinically relevant to the type of contusive injury in humans, in which the dura remains intact. A review study by M Sharif-Alhoseini [20] showed that, of 2087 preclinical model studies of traumatic SCI, contusion injuries were the most common injury model. Regarding the region of contusion, the study also showed that cervical patterns accounted for only 12 percent, compared to thoracic patterns, which accounted for 81 percent. This finding highlights the need to address more cervical injuries in preclinical studies of traumatic spinal cord injury, given that cervical spine injuries are the most common type of traumatic spinal cord injury, particularly in young people.

### 3.2. Traumatic Spinal Cord Injury Downregulates miR133b at the Injury Site, with Dysregulation Limited to Proximal/Distal Parts of the Spinal Cord

In this study, we investigated miR levels at different time points between 30 min and 7 days after SCI. Our studies showed that contusion injury caused a significant early (30 min) upregulation of the endogenous level of miR133b in the area of injury compared to the level measured in the normal cervical cord. This early elevation was also significantly different from the levels measured in the lesion at different timepoints following an SCI. Another finding was that following this initial increase, miR levels gradually decreased and reached the baseline level assessed in uninjured mice by 16 h. The decrease in miR level became more pronounced at 3 days (55% decrease compared to the uninjured group), and by 7 days, the remaining level of miR133b at the lesion site was 20% compared to uninjured spinal cord.

It has been reported in literature that upon spinal cord injury, various types of miRs showed significant changes due to alterations in the expression of different cells, such as neurons and oligodendrocytes [21], as well as the infiltration of the immune cells in the injured area [22,23]. The sustained decrease in the endogenous level of miR133b observed over a period of 7 days might be related to the progression of cell death in miR133b associated cells. The cell type specificity of miR133b in our model and how a moderate SCI would affect the miR133b expression in comparison with a severe type remain to be determined. As for the initial increase in miR133b expression within 30 min post-injury, one possible explanation could be the infiltration of immune and vascular cells, as previously described in other types of miRs [24,25,26,27].

Having determined the dynamics of miR133b in the contused spinal cord, we then continued the investigation of the extent to which a local traumatic injury would affect miR levels in other segments of the spinal cord. For this purpose, we collected the tissue from two different regions of the spinal cord: (1) the rostral part represented by the medulla and C1 and (2) the thoracic part, for which we harvested the tissue from the T5–T7 levels. In contrast to miR levels measured in the contused spinal cord, the dynamics of miR133b in the rostral and caudal parts demonstrated no significant changes compared to normal, uninjured mice. The expression pattern was similar between these two segments, with no notable significant changes in the level of miR133b. Our injury model caused bruising of the dorsal spinal cord with ruptured blood capillaries and partial sparing of tissue at the site of injury. Despite sparing of the ventral spinal tracts ipsilateral to the injury as well as sparing of the contralateral hemicord, the effect of injury was not transmitted and observed in the proximal and distal segments of the spinal cord. One possible explanation is that these segments are predominantly connected by white matter tracts and a remote response to injury would not be induced until higher order synapses occurred at the cortical level.

Our work showing that changes in miR133b post-SCI were limited to the lesion is consistent with another study that reported other miR changes locally at the site of injury over a period of 14 days following a thoracic spine injury. Moreover, the same group reported very limited abnormalities in regions rostral and caudal to the site of injury [28].

### 3.3. Cervical Contusion Injury Affects the Level of miR133b in the Prefrontal Cortex but to a Lesser Extent Than in the Lesion Area

The extent to which contusion injury could alter miR133b levels in the corresponding area of the motor cortex was further investigated. To assess this objective, we used a unilateral contusion injury model that allowed us to compare miR133b dynamics in ipsilateral versus contralateral hemispheres. Although a dorsal column contusion injury may spare the corticospinal tract (CST) axons and anterior horn motor neurons in the area of injury [29,30] and, thus, may not be the most appropriate model for such investigations, we were motivated to determine changes, if any, given that most human injuries are of the contusive type. However, our future studies will aim to determine the effect of SCIs on brain miR133b levels in other models, such as dorsolateral hemisection, which leads to complete destruction of the dorsolateral CST tract compared to contusion injury.

Our data showed that an SCI affects not only the local miR level at the injury site, but also, to a lesser extent, miR levels in the prefrontal cortex. For example, a direct comparison between the lesion and both hemispheres showed that shortly after SCI, there was a significant difference between miR levels in the lesion versus both ipsilateral and contralateral hemispheres. We found that 30 min after SCI, there was an approximately 30% increase in miR in the contralateral side compared to the level measured in the lesion, while the ipsilateral miR level remained unchanged. After 1.5 h, there was an increased trend in both hemispheres that remained unchanged up to 16 h, while there was a decrease in miR levels in the lesion. Interestingly, the effect of the brain injury was more pronounced starting 1 day after SCI and resulted in a significantly increased miR level compared to the normal endogenous level measured in uninjured mice 7 days after SCI. In contrast to the miR level measured in the lesion, which showed a significant reduction between 1 and 7 days post-injury, the miR dynamics in the brain were different, with increased levels visible at these time points.

Relationships between changes in the miR level in the lesion and prefrontal cortex were further examined using Pearson correlation coefficient analysis at 0.5, 1.5 h, 16 h, and 7 days after SCI. Here, we found that at 0.5 h after SCI, there was a relationship between the level of miR in the lesion and in the motor cortex, including a strong positive correlation between lesion and contralateral side and weak negative correlation between lesion and ipsilateral hemisphere. We also found that at 0.5 h there was a strong positive correlation between both hemispheres.

At 1.5 h, miR levels at the lesion site were positively correlated with levels in the prefrontal cortex in both hemispheres, with a stronger effect in the contralateral side. Interestingly, at 16 h, the level of miR in the lesion and both hemispheres reached the baseline level measured in uninjured mice. Our analysis showed that 7 days after SCI, the brain responded to the decrease in miR level at the injured site by increasing the level in the contralateral hemisphere.

The changes in miR133b expression in the prefrontal cortex after a spinal cord injury were not unexpected, given that this brain region contains the sensorimotor cortex, where motor neurons originate. However, the pattern of miR133b expression was indeed intriguing. We speculate that the increase in miR133b level in the contralateral hemisphere at later time points post-SCI (7 days), could come as a compensatory response to damaged cells at the lesion scar. Notably, the Pearson correlation coefficient showed a negative correlation (−0.65) between the miR133b level in the lesion and the miR level in the contralateral hemisphere that further supports this hypothesis. An interesting finding was also observed when we compared miR133b levels between the two hemispheres at the same time point (7 days). This could indicate that the brain might have a different mechanism, wherein one hemisphere (ipsilateral side) responds to changes in the other hemisphere, the affected one (contralateral side). However, further studies are needed to determine the mechanism causing these changes in miR133b expression in the brain after SCI. This could lead to a new finding, in which miR133b plays an important role in neuroplasticity and neuronal recovery following a traumatic injury.

The rodent motor cortex experiences dynamic structural changes following a spinal cord injury, such as severe shrinkage of layer 5 corticospinal neurons as early as two weeks after a lesion at the pyramidal decussation [31,32] or loss of corticospinal neurons [33,34]. Other studies have reported corticospinal sprouting after a thoracic spinal cord injury [35,36]. Depending on the injury model, timing, and location, the response of the motor cortex may be different. Here, we found that after a moderate cervical contusion, miR133b level in the brain is more affected a few days after the injury. Future studies will be aimed at exploring structural changes in the motor cortex after miR133b administration.

### 3.4. Contusion Injury Has Minimal Effect on Lung and Spleen Levels of miR133b

In this study, we found that a cervical contusion injury can cause a slight increase, rather than a decrease, in the level of miR133b in off-targets at different time points. However, our statistical analysis showed that there was no significant difference between injured and uninjured mice. In addition, here we have shown a similar expression pattern between lung and spleen over a period of 7 days post-SCI. We noticed a slight increase in the level of miR133b in the spleen at 7 days that could be related to the infiltration of inflammatory cells from the blood post-injury. However, whether or not these changes in the miR133b expression have any effect on the spleen function remains to be determined.

One of our studies has demonstrated that miR133b administered intravenously reaches the contused spinal cord, suggesting the possibility of bypassing the blood–brain barrier and blood–spinal cord barrier [13]. Furthermore, in a recent study, we have demonstrated that miR133b delivered via intranasal route hours after SCI can be quantified at the lesion scar and this effect was further enhanced when miR133b was co-administered with NEO100 [14]. Intrigued by the latest finding, in this study, we chose the lungs as one of our off-targets for two reasons. First, we will continue to explore our intranasal miR133b therapy in a post-SCI setting. Therefore, understanding the dynamics of miR133b in the lungs, as part of the respiratory system, would be very informative for designing interventions that can target lung therapy. Secondly, SCI typically results in some degree of respiratory dysfunction, where lung function is impaired. Respiratory complications are related to the level at which spinal cord injuries occur. For example, 85% of injuries at the upper cervical segment (C1–C4), 60% at the lower cervical segment (C5–C7), and 65% at the thoracic (T1–T12) levels resulted in deficiencies of the respiratory system as a result of phrenic nerve dysfunction (compromising the diaphragm), which originates between C3–5, and weakened accessory respiratory muscles. Of note, respiratory complications, including poor ventilation and aspiration, are the leading causes of mortality in patients with chronic SCIs.

Several studies have shown changes in the spleen function after spinal cord injury. A study by Wu F reported a significant cellular inflammatory response, such as an increase in infiltrating monocytes and neutrophils in the spleen 72 h after a contusion injury at the 3rd thoracic (T3) level in rats, pointing out the role of the spleen in the pathophysiology of an acute spinal cord injury [37].

### 3.5. Clinical Relevance of miR133b

The current standard of care for acute traumatic spinal cord injury is surgical decompression and stabilization to prevent secondary injuries from ongoing compression or swelling and promoting cord perfusion by increasing the mean arterial pressure. However, surgical decompression does not mitigate the sequelae of the primary injury, including the cascade of neuronal and glial dysregulation, apoptosis, pro-inflammatory state, and fibrosis with irregular axonal regeneration. In addition, although the current clinical practice guidelines from AO Spine recommend surgical interventions within 24 h of the injury to maximize neurologic recovery following an acute cervical spine injury [38], it is not always feasible due to limited access to tertiary care centers with spine surgeons or polytrauma with multi-organ injuries, resulting in patients who are too unstable to undergo operations. Furthermore, literature demonstrates that the benefits of surgical decompression rapidly decline if not performed within 24–36 h following an injury [39]. Our prior studies and the results from this study suggest a potential role for intravenous or intranasal administration of miR133b as early as 16 h and late as 7 days post-SCI to either enhance postoperative recovery or offer medical interventions to patients who may not qualify for timely surgical decompression. Future studies and clinical trials are needed to investigate its safety, efficacy, and feasibility in clinical settings.

### 3.6. Limitations and Future Directions

The data presented here show the effect of a moderate unilateral contusion injury on the endogenous level of miR133b at the site of cervical cord injury and different segments of the spinal cord between 30 min to 7 days post-injury. One limitation of our study is the lack of miRNA-mediated regulation of target gene expression that could validate the decreased functional activity of miR133b after spinal cord injury.

While the results clearly indicate that there is an alteration in the level of miR133b in the lesion scar, future studies are important to also determine miR133b changes in body fluids, such as plasma and cerebrospinal fluid (CSF). These approaches will help gain more insight into the relationship between changes in the contused area and how these changes can be quantified systemically. This could open another door where miR133b can be used as a biomarker for traumatic brain and spinal cord injuries.

The injury model used in this study was a moderate contusion injury at the cervical level. However, it will be important to determine the extent to which a severe contusion injury (a) accelerates the decline in miR133b expression and (b) which cells in the injury scar are primarily responsible for miR133b dysfunction.

## 4. Methods

### 4.1. Experimental Design

All animal procedures were approved by the Institutional Animal Care and Use Committee (IACUC, Protocol # 20306) of the University of Southern California, Los Angeles. A total of thirty-five C57BL6 female mice (Charles River, https://www.criver.com, accessed on 15 November 2023) between 6 and 8 weeks of age and weighing 20–25 g at the beginning of the experiment were used in this study. All procedures were conducted following the US National Institute of Health (NIH) guide for care and animal use of laboratory animals.

### 4.2. Spinal Cord Surgical Procedures

The spinal cord injury model used in this study was a moderate severity (80 kilodyne force) unilateral contusion injury at the 5th cervical (C5) level, as previously described (Danilov, 2020). Mice were deeply anesthetized through intraperitoneal injection of a cocktail of ketamine (100 mg/kg) and xylazine (10 mg/kg), and a dorsal laminectomy was performed at C5. The contusion injury was created using the Infinite Horizon (IH) device platform IH-0400 (Precision Systems and Instrumentation, LLC, Fairfax, VA, USA). To induce the injury, the tip of the impactor was positioned on one side of the cord, creating unilateral deficits to the corresponding forelimb. Post-SCI, mice received an injection of Ringer’s lactate solution (1 mL/20 g, SubQ) for hydration, enrofloxacin (2.5 mg/kg, SubQ) for prophylactic treatment against urinary tract infection, and one single dose of Ethiqa XR (3.25 mg/kg, SubQ) for analgesia.

### 4.3. Tissue Collection and miRNA Extraction

The endogenous level of miR133b was assessed in different tissue samples between 30 min and 7 days post-SCI, as illustrated in Figure 1. At the indicated time points, tissue was collected on dry ice and stored at −80 °C for further analysis.

microRNA was extracted from the following tissue samples: (a) 4th cervical (C4)–6th cervical (C6) spinal cord segments containing the lesion, (b) medulla–1st cervical (C1) segment (rostral part), (c) 5th thoracic (T5)–7th thoracic (T7) (caudal part), (d) spleen and lung (off-targets), and (e) pre-frontal cortex (ipsilateral and contralateral parts) using mirVana miRNA Isolation Kit (Invitrogen, Carlsbad, CA, USA, Cat # AM1561). Tissue samples were homogenized and lysed in a denaturing lysis solution that stabilizes RNA and inactivates RNases. Next, samples were subjected to acid: phenol: chloroform RNA extraction according to the manufacturers’ instructions. MicroRNA purification was performed by using the glass fiber filters procedure, followed by exposures to 25% and 55% ethanol to immobilize microRNA. This microRNA was further washed with two different miRNA wash solutions and eluted in a low-ionic-strength solution. miR133b quantification was a two-step procedure that included reverse transcription (RT) and polymerase chain reaction (PCR). In the first reaction, miRNA samples (150 ng/µL) were reverse-transcribed using TaqMan^TM^ MicroRNA Reverse Transcription Kit (ThermoFisher Scientific, Waltham, MA, USA, Cat # 4366596). Real-time PCR reactions were assessed using TaqMan^TM^ Universal Master Mix II, no UNG (ThermoFisher Scientific, Waltham, MA, USA, Cat # 4440043), TaqMan assay for miR133b (Applied Biosystems, Waltham, MA, USA, Cat # 4440886), and TaqMan Control miRNA assay for U6 snRNA (Applied Biosystems, Waltham, MA, USA, Cat # 4427975. The level of miR133b was assessed by RT-qPCR using TaqMan^TM^ Universal Master Mix II, no UNG (ThermoFisher Scientific, Waltham, MA, USA, Cat # 4440043), TaqMan assay for miR133b (Applied Biosystems, Waltham, MA, USA, Cat # 4440886), and TaqMan Control miRNA assay for U6 snRNA (Applied Biosystems, Waltham, MA, USA, Cat # 4427975). To ensure accurate data interpretation, the expression level of miR133b was normalized to U6 snRNA expression, which remained unaffected following our experimental factors. For both miR133b and U6 snRNA, the concentration of total cDNA was the same in all the samples analyzed. The quantitation of miR133b was estimated based on C_T_ (cycle threshold) values. The ∆C_T_ was calculated by subtracting the C_T_ of the control (U6 snRNA) from the C_T_ value of the miR133b. The ∆∆C_T_ was calculated by subtracting the ∆C_T_ of control (uninjured sample) with the ∆C_T_ of the test (injured sample). The fold change was generated using the equation 2^−∆∆CT^.

### 4.4. Statistical Analysis

Data from different independent experiments were analyzed using GraphPad Prism version 9 software. The results were plotted as mean ± standard error of the mean. Statistical analysis was performed using a parametric test, such as one-way analysis of variance (ANOVA) because of its robustness. The Tukey’s multiple comparison test was used to assess the difference among different groups and Dunnett’s post hoc test to determine the statistical difference between samples and control. Pearson correlation coefficient was employed to study the association between three or more variables. A *p*-value of less than 0.05 was considered statistically significant.

## 5. Conclusions

The studies presented here investigated the effect of moderate contusion injury on the endogenous level of miR133b in the cervical cord lesion site, rostral and caudal segments to the injury site, brain, and off-targets over a period of 7 days post-injury. Our data demonstrated that (a) most changes were restricted to the injured area with very limited miR133b dysregulation in the rostral and caudal parts to the injured site; (b) SCI caused changes in the endogenous level of miR133b in the motor cortex, especially days post-injury; (c) there was specificity in the miR133b response between ipsilateral and contralateral hemispheres after SCI in mice. In this study, we provide evidence of miR133b dysfunction after SCI, prompting future studies to explore (a) a better timing of intervention before miR133b levels become significantly reduced and (b) a different duration of exogenous miR133b therapy that might extend up to 7 days post-SCI. Moreover, our findings using a contusive spinal cord injury model could be tested in other injury models, such cortical contusion brain injury. In fact, our strategy of intranasal co-administration of miR133b in a NEO100-based formulation showed great potential in delivering other types of miRs, not only to the contused spinal cord but also to the injured brain.

Although this study was performed in young female mice, future studies can be designed to determine the impact of gender and aging in the regulation of endogenous miR level post-SCI. Future functional analysis studies will be required to explore the regenerative potential of miR133b when applied at a time point earlier than 24 h after spinal cord injury.

## Data Availability

The data presented in this study are available on request from the corresponding author.
